# The Quality of Life among Siblings of Autistic Individuals: A Scoping Review

**DOI:** 10.3390/jcm12030735

**Published:** 2023-01-17

**Authors:** Giuseppe Quatrosi, Dario Genovese, Emanuele Amodio, Gabriele Tripi

**Affiliations:** 1 Department of Psychology, Educational Science and Human Movement, University of Palermo, 90128 Palermo, Italy; 2 Department of Health Promotion, Mother and Child Care, Internal Medicine and Medical Specialties (PROMISE), University of Palermo, Via del Vespro, 133, 90127 Palermo, Italy; 3 School of Specialization in Child Neuropsychiatry, University of Palermo, 90128 Palermo, Italy

**Keywords:** siblings, autism spectrum disorders, autism, quality of life, QoL, well-being

## Abstract

Autism spectrum disorders are a heterogeneous group of neurodevelopmental disorders presenting at a tender age, defined by qualitative deficits in social interactions and communication, thus having a substantial influence on the subject’s family unit. Quality of life (QoL) refers to a person’s perspective of their life situation, cultural and value background, objectives, expectations, and standards. When focusing on childhood and adolescence, age-related changes should be considered. For this scoping review, the authors used three peer-review literature database sources (PubMed/MEDLINE, Scopus, and ERIC) to delve deeper into components of the QoL of non-autistic siblings of autistic individuals. At the completion of the eligibility phase, 9 studies were included out of the 96 initial records. A total of 4/9 articles (45%) compared the non-autistic siblings of autistic individuals to the siblings of non-autistic people, while 3/9 articles (33%) compared the first group to the non-autistic siblings of patients with other chronic diseases. A total of 5/9 studies adopted specific questionnaires to evaluate QoL. Results from 6/9 articles revealed that the autism condition has varying effects on non-autistic siblings’ QoL. According to the considered research, non-autistic siblings of autistic individuals experienced decreased psychological well-being, less perceived social support, increased aggressiveness and conflict-proneness, and higher levels of anxiety and stress impacting their QoL. The present findings provide important implications for additional and more punctual studies in this sector. Furthermore, as being a non-autistic sibling of an autistic individual is commonly undervalued, this review advocates the need to organize and improve support services for siblings.

## 1. Introduction

Autism spectrum disorders (ASD) are defined as a complex group of neurodevelopmental disorders with onset in the first three years of life, characterized by qualitative impairments of social interactions, communication, and a limited, stereotyped, and repetitive repertoire of interests and activities [1]. The autism spectrum is characterized by considerable clinical heterogeneity, although patients share some fundamental characteristics of the diagnosis, such as, in the first place, “social withdrawal”. This heterogeneousness reflects on specific autistic characteristics, developmental trajectories, age and onset, the course of the neurodiversity, and the variability of the impairment in communication, motricity, or language [2,3,4,5]. Over the past decade, an increase in the prevalence of autism spectrum disorder has been described globally, with wide geographical variability. This increase reflects both a greater awareness of society about the disorder and advances in the diagnosis and definition of cases [6]. The autism condition is more commonly diagnosed in family members of people who have already been diagnosed, suggesting a strong genetic basis for the neurodiversity and other causes [7,8]. Autism has a significant impact on the family unit of the individual, whether it is the parents or siblings of the autistic person. It is not uncommon to find in parents of autistic children with higher levels of stress and depression and a perception of less social and family support when compared with the findings in parents of non-autistic youths or children with other disabilities [9,10,11,12]. Moreover, parents of autistic children show concerns about the problems related to the social and communication deficits of their autistic children, as well as about the repercussions on the domestic environment and on their other non-autistic children, to whom less time and energy can often be dedicated [13,14,15].

According to the World Health Organization (WHO), quality of life (QoL) can be defined as a subject’s perception of his or her position in life, cultural and value context, goals, expectations, and standards [16]. When QoL focuses on childhood and adolescence, age-related alterations should be considered. In fact, the perception of what QoL is for a child seems to be different than for an adult. In childhood, environmental and economic conditions appear to have a greater impact on the quality of life than the physical or psychological condition of the subject [17].

In 1994, Cicirelli [18] describes the relationship between siblings as the one that lasts longer than all human relationships, which makes it unique. However, this link appears to change over time. Although the bond between siblings appears to be particularly close in childhood, in the general population it is usual to find a lower satisfaction with the bond between siblings during adolescence and in general a decrease in contact in early adulthood, in coincidence with the establishment of their independent life. By contrast, siblings appear to share greater satisfaction and interaction during mid and late adulthood.

In a study conducted on families of autistic children by Ward et al. [19], participants recognized both the difficulties and positive aspects of living with a young autistic person. This perception appears to take on different nuances depending on the age and sex of the siblings. Therefore, conflicting feelings appear to coexist in these subjects: the younger ones appear more linked to experiences and play, and the older ones more frequently report a greater sense of responsibility, protection, and a greater inclination to empathy and introspection following the sibling’s autism diagnosis. Brothers often mentioned the aggressive behaviors of the child/adolescent and the desire to have more opportunities to play with the autistic sibling, while sisters reported more of the relational and communicative difficulties of the young autistic sibling.

In a recent Greek study carried out by Koukouriki et al. [20], the anxiety of siblings of autistic children was significantly associated with parental anxiety independent of parental perceived social support and demographic factors, while the health-related quality of life (HRQOL) of siblings of autistic children was associated with perceived social support independent of the physical and psychological health of parents and demographic factors.

In childhood and adolescence, siblings of autistic children may be at greater risk of social and behavioral adaptation problems. Although an estrangement in adulthood is described, the QoL results of siblings of autistic people are warning signs that should be taken seriously, as these same siblings could potentially inherit long-term family care responsibility from their parents. In general, it seems desirable that there will be a greater number of age-specific research on these subjects in the future [21,22].

Recent studies focus instead on the positive protective factors that impact the family after the diagnosis of autism in one of the children, such as greater family support or the development of a greater capacity for introspection and empathy in the family members of the autistic person [9,23].

In 2007, in a review of the literature on typical siblings who have a brother or sister with a neurodiversity, Schuntermann [24] identified six broad domains. The first focuses on family systems that seem relevant to assessing the relational functioning of siblings within their families. Four other domains address specific aspects of experiences shared between siblings. These include parent–child triadic interactions, sibling relationships, sibling settings and siblings’ intergenerational settings (grandparents), and siblings’ social settings (friends, peers). The sixth domain focuses on the perspectives of brothers on how to give meaning to life with a brother or sister with developmental problems, now and over time.

This review, therefore, wants to deepen the aspects regarding the quality of life of the siblings of autistic people in relation to childhood and adolescence, with attention to the relational dynamics existing in families, the protective and risk factors, as well as the possible methods of evaluation and intervention on these individuals.

## 2. Materials and Methods

Scoping reviews are considered a valid approach for synthesizing health evidence, especially when a systematic approach cannot be undertaken [25]. A scoping study may be beneficial when there are gaps in the existing scientific literature: in fact, it helps to identify the latter and to examine what has been found via the analysis of experts’ research activity [26]. For this reason, the research group chose to adopt the Khalil et al. evidence-based approach to scoping reviews, based on Arksey and O’Malley’s methodological framework [27,28]. The preferred reporting items for systematic reviews and meta-analyses extension for scoping reviews (PRISMA-ScR) guidelines were used to report the process and the results [29].

### 2.1. Research Questions

The authors attempted to answer the following questions:

1. How does autism affect the quality of life of non-autistic siblings of autistic individuals?
2. Does the QoL of the siblings of autistic people differ among the different age groups (infancy, adolescence, adulthood)?
3. Were specifically validated tools used to assess the quality of life of non-autistic siblings of autistic individuals? Alternatively, which methods have been used?

### 2.2. Search Strategy

For the present scoping review, we consulted three peer-review literature database sources, namely PubMed/MEDLINE, Scopus, and ERIC. The literature research was conducted on 1 July 2022 by combining free text words and medical subject headings (MeSH). The search strategy consisted of a combination of general autism terms, QoL terms, and the condition of being a sibling. The following search string was finally used for the study purpose:

((("Autism Spectrum Disorder*" OR ASD OR "Autism Spectrum" OR Autism) AND ("Quality of life" OR QoL OR "Life Quality" OR "Health-Related Quality of Life" OR "Health Related Quality of Life" OR HRQoL)) AND (Sibling* OR Brothers OR Sisters))

### 2.3. Study Selection

The search string allowed the authors to identify 136 research articles, reduced to 96 after the removal of the duplicates. Duplicates were removed profiting from Zotero ver. 6.0.10.

Inclusion criteria were set with the scope to select peer-reviewed reports assessing the quality of life of the non-autistic siblings of autistic people. The authors included each article:

- Written in the English language;
- Assessing the quality of life of siblings of autistic individuals;
- In which a QoL-validated questionnaire was administered or, alternatively, a questionnaire investigating specific aspects of the QoL.

Inclusion criteria were applied both in the screening and eligibility phases. As per the screening stage, inclusion criteria were applied to the title and the abstract of each identified article, whereas during the eligibility stage, they were applied to the full text of the screened articles.

The authors excluded studies in which neither quality of life instruments nor specific information for non-autistic siblings were mentioned or applied. It was excluded from the scoping review each article not written in the English language, as well as all the reviews, commentaries, book chapters, case reports, and studies adopting a qualitative research design.

### 2.4. Charting the Data

A “descriptive analytical” method was used to extract all the relevant information, as per Arksey and O’Malley’s process suggestion [28]. In detail, from each article, we collected: 1. The article’s first author and relative reference; 2. The title of the article; 3. The year of publication; 4. The country in which the study was conducted; 5. The design of the study; 6. The purpose of the study; 7. The number of participants; 8. The age range; 9. The quality of life tools; 10. The respondent type; 11. The outcomes; 12. The main findings.

### 2.5. Collating, Summarizing, and Reporting the Results

Data were collated and summarized using as a scheme the items previously presented in the “Charting the Data” section.

## 3. Results

### 3.1. The Literature Search

**Figure 1:**
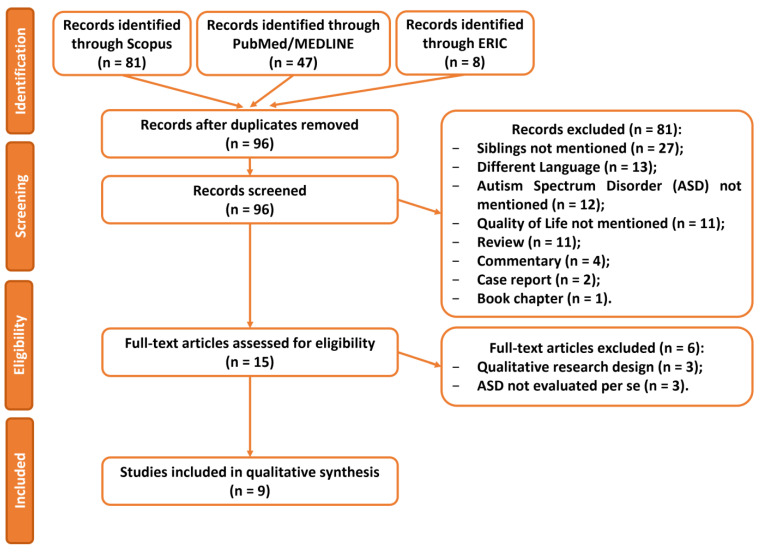
Flowchart representing PRISMA flow diagram of studies’ screening and selection.

As reported in Figure 1, in the identification phase, a total of 136 records were detected through the Scopus (81), PubMed/MEDLINE (47), and ERIC (8) searching platforms; among them, there were 40 duplicates, which were removed.

From the 96 remaining records, 81 were excluded after the screening phase for the following reasons: 27 articles did not mention any relationship between the autistic person and a sibling (33%); 13 records were not written in English (16%); 12 reports did not mention autism (15%); 11 articles did not aim to assess the quality of life of their sample (14%); the remaining 18 records were reviews (11; 14%), commentaries (4; 5%), case reports (2; 2%), or book chapters (1; 1%).

At this point, 15 full-text articles were assessed for eligibility, and 6 of them were excluded for the following criteria: 3 were qualitative studies (50%) and the remaining 3 did not evaluate the burden of autism per se (50%).

At the end of the eligibility phase, 9 studies were included in the scoping review [9,20,22,30,31,32,33,34,35].

### 3.2. Characteristics of the Included Studies

Table 1 summarizes the characteristics of the nine articles included by the researchers. Overall, 7/9 reports (78%) adopted a case-control study design [9,20,30,32,33,34,35], while the remaining 2/9 articles (22%) decided to perform a cross-sectional study [22,31].

As per the country, 3/9 reports (33%) were carried out in a European country (in particular, one article (11%) in the United Kingdom [32], another one (11%) in Greece [20], and the last one (11%) in Spain [9]); 2/9 articles (22%) were conducted in Brazil [30,31]; 2/9 records (22%) were directed in the United States of America [22,34]; the remaining 2/9 studies (22%) were conducted in Asia (namely, one article (11%) in China [33] and the other one (11%) in Iran [35]).

All the articles were published after 2000.

**Table 1:** Characteristics of the nine articles included by the researchers.

Reference Article [Reference No.]	Title	Publication Year	Country	Study Design
Ferreira Marciano, A.R. [30]	Quality of life in siblings of autistic patients	2004	Brazil	Case-control
Orsmond, G.I. [22]	Siblings of individuals with an autism spectrum disorder: Sibling relationships and well-being in adolescence and adulthood	2009	United States of America	Cross-sectional
Vieira, C.B.M. [31]	Quality of life of siblings of children included in the autism spectrum	2012	Brazil	Cross-sectional
Hastings, R.P. [32]	Self-reported behavior problems and sibling relationship quality by siblings of children with autism spectrum disorder	2013	United Kingdom	Case-control
Chan, J.Y.N. [33]	Psychological adjustment of siblings of children with autism spectrum disorder in Hong Kong	2016	China	Case-control
Tomeny, T.S. [34]	Sibling relationship quality and psychosocial outcomes among adult siblings of individuals with autism spectrum disorder and individuals with intellectual disability without autism	2017	United States of America	Case-control
Esfahani, F.N. [35]	Internalizing and externalizing problems, empathy quotient, and systemizing quotient in 4- to 11-year-old siblings of children with autistic spectrum disorder compared to control group	2018	Iran	Case-control
Koukouriki, E. [20]	Self-reported health-related quality of life (HRQOL) and anxiety among Greek school-age siblings of Iindividuals with autism spectrum disorders (ASD) in relation to parental mental health and social support	2020	Greece	Case-control
Garrido, D. [9]	Siblings of children with autism spectrum disorders: social support and family quality of life	2020	Spain	Case-control

Table 2 shows all the relevant information extrapolated from each included article.

Looking more in-depth: 4/9 reports (45%) compared the aforementioned brothers and/or sisters to the siblings of people who were also non-autistic [9,20,32,33]; 3/9 articles (33%) compared the non-autistic siblings of autistic individuals to the non-autistic siblings of patients with other chronic diseases [30,34,35]; of the remaining 2 articles, one (11%) compared adolescent siblings of autistic individuals to adult ones [22], while the other one (11%) evaluated the QoL of the abovementioned siblings without including a control-contrast group [31]. Since only 54% of the studies included in this review employed specific indicators to assess QoL [9,20,30,31,33], results were presented in two distinct subsections.

### 3.3. Aims of the Included Studies and Types of Questionnaires Administered

Ferreira Marciano and Scheuer [30] evaluated the QoL among non-autistic siblings of autistic patients through the comparison with siblings of individuals with specific speech articulation disorders. Both cases and controls were aged between 7 and 11. Their QoL was quantified by administering the Autoquestionnaire Qualité de Vie Enfant Imagé (AUQEI—Quality of Life of Children aged 4 to 12 years) [36].

Orsmond et al. [22] investigated the well-being of siblings of autistic individuals among two different age groups, adolescents (subjects with an age comprised between 12 and 18) and adults (individuals between 19 and 55 years of age). The items that may variously compose a QoL questionnaire were singularly assessed through the distribution of several tools, namely: the Instrumental Involvement—Caregiving [37,38], the Positive Affect Index (PAI) [39], the Center for Epidemiologic Studies Depression Scale (CES-D) [40], eight scales from the COPE [41], the Perceived Social Support Scale [42], and Scales of Independent Behavior-Revised (SIB-R) [43].

Vieira and Fernandes [31] aimed to evaluate the QoL of siblings of autistic children via their self-response to the World Health Organization Quality of Life (WHOQoL)-BREF questionnaire [16,44]. The abovementioned siblings were in their early adulthood, aged between 16 and 30.

Hastings and Petalas [32] collected the self-reports compiled by siblings of autistic children pertinent to their behavior problems, comparing these self-reports to a self-report normative sample composed of children from the general population. The two groups were matched per age, enrolling all the children between 7 and 17 years of age. Mothers compiled the parent version of the Strengths and Difficulties Questionnaire (SDQ) [45], while the siblings fulfilled two questionnaires: the self-report version of the SDQ (exclusively children 11–17 years of age), and the Sibling Relationship Questionnaire-Revised (SRQ-R) [46].

In a study by Chan and Lai [33], parents of children between 6 and 18 years old were asked to compile the following tools: Strengths and Difficulties Questionnaire (SDQ) [45], General Health Questionnaire (GHQ-12) [47], WHOQoL-BREF [16], and General Functioning Scale of the Family Assessment Device (FAD) [48]. Siblings completed the Sibling Stress Scale (SSS).

Tomeny and colleagues [34] aimed to assess the adult sibling relationships in families of individuals with a developmental disability; therefore, the authors decided to gather information from the 18- to 62-years-of-age siblings of autistic individuals and the 18- to 52-years-of-age siblings of patients with an intellectual disability. The siblings were asked to fulfil three questionnaires: Depression, Anxiety, and Stress Scale (DASS) [49], Lifespan Sibling Relationship Scale (LSRS) [50], and Satisfaction with Life Scale (SWLS) [51].

Esfahani et al. [35] tried to individuate the problems of living with an autistic sibling to augment their QoL. To do so, the researchers chose to assess the same item among two groups: the first was composed of siblings of autistic children, whereas the second was composed of siblings of patients with a chronic disease. The samples were matched per age, selecting siblings between 4 and 11. Each sibling completed three scales: the Gilliam Autism Rating Scale-Second Edition (GARS-2) [52], the Empathizing-systemizing test, and the Child Behavior Checklist [53].

Koukouriki and Soulis [20] aimed to assess if there were associations between siblings’ HRQoL or anxiety and parental psychological health, perceived social support, and other demographic factors. The authors chose to enroll children whose age was between 8 and 18, being siblings of autistic people persons (case) or siblings of non-autistic children (control). They were administered several questionnaires, namely: the Health-Related QoL (KIDSCREEN-27) [54], the State-Trait Anxiety Inventory for Children (STAIC, A-Trait) [55], the General Health Questionnaire (GHQ-28) [56], and the Multidimensional Scale of Perceived Social Support (MSPSS) [57].

Finally, Garrido et al. [9] tried to evaluate the possible influence of being a non-autistic sibling of an autistic child on the family QoL. To do so, the authors chose to compare the abovementioned siblings aged 6–12 to a comparison group composed of siblings of non-autistic children, matched per age. Parents were asked to complete the following measures: the Social Communication Questionnaire (SCQ) [58], the Structural Social Support [59], and the family QoL of People Survey (FQoLS) [60]. Sibling completed the following questionnaires: the comprehension test of grammatical structures (CEG) [61], the Peabody Picture Vocabulary Test (PPVT-III) [62], the Clinical Evaluation of Language Fundamentals-Fourth Edition (CELF-4) [63], the Movement Assessment Battery for Children-Second Edition (MABC-2) [64], and the Wechsler Intelligence Scale for Children (WISC-IV) [65].

### 3.4. Assessment of the Quality of Life of Non-Autistic Siblings of Autistic Individuals

With reference to the age range of the population sample analyzed, 2/5 articles (40%) specifically evaluated the QoL of infant and/or adolescent siblings [20,30], whereas one record (20%) assessed the QoL of siblings in their adulthood [22,31]. The remaining two articles evaluated the family QoL or the parents’ quality of life [9,33].

QoL was self-reported in each study; additionally, in three of them (60%), one or both parents reported siblings’ perceived quality of life [9,20,33]. The quality of life resulted in being significantly impaired in 4/5 articles (80%) [9,20,30,33], whereas it was not meaningfully reduced in the remaining report [31]. Unfortunately, the QoL of non-autistic siblings of autistic individuals was diversely assessed among the included studies. For this reason, it is worth analyzing each report singularly.

Ferreira Marciano and Scheuer [30] found that the QoL among the non-autistic siblings of autistic individuals was impaired, being significantly worse than the QoL of the siblings of patients with a speech disorder. This result is confirmed by Koukouriki and Soulis [20]: after controlling for sex and age of the children, the HRQoL of the siblings of autistic individuals resulted in being lower than in the control group. Perceived social support from the family was shown as a predictor of HRQoL of the siblings of autistic children. Notably, the cases group had lower scores than the control group in each subscale of KIDSCREEN-27, with the highest impairment in the subscale of psychological well-being. The hierarchical regression models showed that social support from the family was statistically significantly associated with siblings’ HRQoL.

Contrarily, Vieira and Fernandes [31] found that the least satisfying domain was the environment one; the latter was significantly impaired when compared to the physical and psychological domains. Nevertheless, it was noticed that the self-reported QoL for the analyzed sample was not significantly impaired.

Finally, the results of the Chan and Lai [33] study showed that parents’ QoL was lower than that of community populations. Similarly, Garrido et al. [9] found statistically significant differences in the severity of the autism condition and in the FQoL scores of the siblings of autistic people, when compared to the siblings of non-autistic children. Another important result is that perceived social support might be a predictor of FQoL; in particular, the higher the perceived social support, the higher the quality of life.

### 3.5. Assessment of Well-Being and Psychological Health-Related Findings

Referring to the age range of the population sample analyzed, 5/7 of the articles (71%) evaluated the well-being and the perceived psychological health of infant and/or adolescent siblings [9,20,32,33,35], while one research study assessed the same items during their adulthood [34]; the remaining article, as previously stated, compared the adolescent siblings to the adult counterpart [22]. The researchers decided to examine the results separately since, similar to the preceding subsection scenario, varying approaches were used to establish the aforementioned associations.

#### 3.5.1. Depression, Anxiety, and Stress

Overall, 5/7 of the reports (71%) assessed the presence and severity of depression, anxiety, and stress among the non-autistic siblings and their families [20,22,33,34,35].

Koukouriki and Soulis [20] discovered that the anxiety levels of the case group were significantly higher than the ones in the group of the non-autistic children’s siblings. Moreover, the parents of the cases group showed higher levels of psychological distress than the control group. The hierarchical regression models showed that the anxiety levels of siblings of autistic children were significantly associated with parental anxiety. These findings are supported by Chan and Lai [33], who found that family stress could be caused by several endorsed experiences. As a result of their investigation, Tomeny et al. [34] found that siblings of autistic individuals are at a greater risk of developing depression and stress when compared to siblings of people with an intellectual disability.

Esfahani et al. [35], in reference to gender differences within the group of siblings of autistic people, found that the sisters had higher scores than their male counterparts in terms of anxiety and depression, suggesting different attitudes between females and males.

Surprisingly, Orsmond et al. [22] found no statistically significant difference in terms of the presence of depressive symptoms between adolescent siblings and their adult counterparts; in both groups, depression was not detected. No gender differences were found, as well.

#### 3.5.2. Behavioral Attitudes and Associated Factors

Hastings and Petalas [32] did not find a significant elevation in behavioral and emotional problems among siblings of autistic children. There was some indication that siblings’ relationships were associated with the behavior problems of the autistic child; finally, it was found that higher levels of behavior problems were associated with lower levels of warmth/closeness and with an increased conflict attitude.

Esfahani et al. [35] found that the aggressive behavior of the siblings of autistic individuals resulted in being statistically significantly higher than the same item among the siblings of people with chronic disease. The externalizing score was significantly higher in the first group than in the control group.

Finally, Tomeny et al. [34] found that siblings of autistic individuals may show lesser positive attitudes about their relationship with their brother/sister when compared to siblings of people with an intellectual disability; they could provide less support to their autistic siblings due to fewer positive sibling relationship attitudes.

#### 3.5.3. Perceived Social Support, Siblings’ Perceived Role, and Siblings’ Adjustment

Chan and Lai [33], other than assessing the parents’ QoL of distinct groups of children aged between 6 and 18, surveyed the psychological adjustment of siblings of autistic children through the comparison with a normative sample from the general population. The findings revealed that total difficulties rates were higher among the reports of parents of autistic children than in the ones of parents of non-autistic children. Siblings’ adjustment was predicted by family stresses, other than by parents’ QoL, but not by age, gender, or the birth order of the sibling. Nevertheless, the scores of the reports completed by the parents were not statistically different between the two groups, except for some concerns about peer relationships and prosocial behaviors among siblings of autistic children.

Garrido et al. [9] aimed at assessing which factors may determine the impact of having an older autistic sibling on several developmental domains. The results suggested statistically significant differences in the severity of autism and perceived social support between the group of siblings of autistic people and the siblings of non-autistic children.

Finally, Orsmond et al. [22] found that, even if adolescent siblings reported greater social support than adult ones, both groups experienced a similar degree of positive affects in the relationship with their autistic brother or sister. In terms of developmental trends, there were different coping strategies: adolescents used more emotion-focused coping strategies than adults, who adopted more problem-focused coping strategies. There was no gender difference among adolescent siblings, while it was noticed that adult sisters of autistic sisters showed more engagement in shared activities than adult brothers of autistic sisters.

## 4. Discussion

### 4.1. How Does Autism Affect the Quality of Life of Non-Autistic Siblings of Autistic Individuals?

The main objective of this scoping review was to determine the impact of the autism spectrum on the quality of life of the non-autistic siblings of autistic people. The QoL was either evaluated with appropriate tools or, indirectly, through the administration of distinct questionnaires ferreting the siblings’ well-being and their perceived psychological health. The results showed that the autism condition variously impacted the quality of life of non-autistic siblings in 6/9 included studies [9,20,30,33,34,35]. Of the remaining 3 studies, the one conducted by Orsmond, Kuo, and Seltzer [22] analyzed sibling relationship patterns in adolescence and in adulthood, while the remaining 2/9 did not find a statistically significant difference between the QoL of the non-autistic siblings of autistic people and the control groups [31,32]. The studies taken into account show that non-autistic siblings of autistic people suffered the effects of a negative impact on the quality of life with increased aggressivity and conflict-proneness [32,35], increased anxiety and stress [20,33,34], lower positive attitudes regarding the relationship with the brother or sister affected by the disorder [34], reduced psychological well-being [20], and less perceived social support [9,20].

Indeed, several studies suggested that growing up in a family with an autistic person could lead to a greater burden of responsibility, as well as concerns about the future of the sibling and the role that will have to be assumed in the future once the parents will not be able to assist the affected sibling or after their loss [19,23,66,67,68,69,70]. Non-autistic siblings of autistic people could also prevail feelings of frustration dictated by the perception of a greater parental attention toward the affected sibling, due to the numerous needs of the autistic child, as well because of the possible burden of parental distress [66,67,71,72].

Moreover, these children often encounter difficulties as it is particularly complex both to explain to people who are outside the nuclear family the condition of disability of the sibling, sometimes not clearly perceived or visible by others, and to make people understand their struggles regarding their experience within the family; as a consequence, non-autistic children are frequently reluctant to talk about their autistic sibling [66,70]. It is worth noting that Moyson and Roeyers [67] have defined autism as an “invisible disability” since normal physical appearance and behavior conceal the disorder that lies beneath [67]. A study conducted by Skär [73] revealed that the word “disabled” is commonly associated with the usage of technical devices (such as wheelchairs), underlining the common thought that they are a prerequisite for people with disabilities. Differently from a visible disability, children may not fully comprehend the condition of an autistic person and, thus, be suspicious of the “non-perceivable” disability. This last point is relevant in terms of perceived social support that in non-autistic siblings of autistic people appears considerably reduced when compared to the control groups.

Despite the potential problems related to having an autistic sibling, non-autistic siblings are often concerned about their siblings’ well-being and possibilities for social inclusion [67,70,74] and, according to Orsmond et al. [22], the relationship between the non-autistic and autistic siblings resulted in being stable, both in adolescence and in adulthood.

### 4.2. Does the QoL of the Siblings of Autistic People Differ among the Different Age Groups (Infancy, Adolescence, Adulthood)?

Peculiarities have emerged regarding the different ages of siblings, allowing the authors to affirm that in childhood and adolescence aggressivity, proneness to conflict, anxiety, and stress may be increased and that levels of perceived social support appear to be lower when compared with the control groups of their peers [9,20,33,35]. At the same time, among adult non-autistic siblings of autistic people, Tomeny et al. [34] found that they may show lesser positive attitudes about their relationship with their siblings when compared to siblings of people with an intellectual disability; this tendency may drive toward the development of depressive and anxious symptomatology [34]. In the study of Orsmond et al. [22], adolescents reported more social support than adults did. The age might also have a different weight on the type of coping mechanism adopted. In fact, adolescents tended to use coping mechanisms based on emotions, while the adults’ one was based on problem-solving [22], which could be read as a more mature coping strategy; these findings are consistent with the scientific literature [75,76]. It should be emphasized that the various studies address the differences relating to the various age groups using non-comparable assessment tools and therefore some of the impairment aspects that emerged in certain age groups may not be age specific.

### 4.3. Were Specifically Validated Tools Used to Assess the Quality of Life of Non-Autistic Siblings of Autistic Individuals? Alternatively, Which Methods Have Been Used?

The tools used for the quality-of-life assessment are summarized in Table 2. Among the studies of this scoping review, 5/9 used specific tools to assess the QoL [9,20,30,31,33]. In two studies [31,33] the WHOQoL-BREF questionnaire was administerd. Ferreira Marciano et al. [30] assessed the QoL of non-autistic siblings of autistic people using the AUQEI questionnaire, specific for children aged 4 to 12 years. Koukouriki et al. [20] administered the KIDSCREEN-27, a Health-Related QoL questionnaire. Finally, Garrido et al. [9] assessed the family quality of life by using the Family QoL of People Survey (FQoLS).

The scarcity of studies, combined with the profound heterogeneity of the tools used, resulted in the inclusion of studies that, while not using specific QoL assessment tools, had the goal of analyzing it by the administration of various self-reported and parent-proxy reported questionnaires that investigated specific items (4/9 studies) [22,32,34,35].

## 5. Conclusions

The current scoping review revealed significant implications for future research as well as the necessity to organize and enhance siblings’ support services. In fact, the condition of being a non-autistic sibling of an autistic individual is frequently underestimated, and there is no agreement on the methodologies for measuring these individuals’ living conditions in the scientific literature.

Nonetheless, this review encountered several limitations. First, different QoL tools were employed throughout the included studies, in addition to the different control groups and age ranges. Even among the research that used a QoL questionnaire, there was a general lack of consistency: only two reports used the WHOQoL-BREF, while the remaining three used different tools. Furthermore, many studies lacked appropriate sampling methods to address the research goal(s). Indeed, in five of the nine publications, the researchers did not reach a total sample size of 100 people, limiting the robustness of the conclusions and their generalizability. The language of autism is undergoing rapid and multiple changes. The authors’ intention in this review, in accordance with the paper “Avoiding ableist language: Suggestions for autism researchers” by Bottema-Beutel et al. [77], was to advocate an identity-first language and to avoid ableist language.

By contrast, these findings provide useful considerations for further and more punctual research in this field. Indeed, there is a need to develop a method that is consistent in evaluating QoL in non-autistic siblings of autistic individuals. Aside from a better grasp of how to evaluate QoL in these patients, it appears to be of interest how to assess the overall burden that siblings are suffering, as well as therapeutic possibilities.

## Figures and Tables

**Table 2:** Relevant information extrapolated from each included article.

Reference Article [Reference No.]	Purpose of the Study	No of Participants	Age Range	Tools	Respondent Type	Outcomes	Main Findings
Ferreira Marciano, A.R. [30]	To evaluate the quality of life (QoL) among siblings of autistic people	− Siblings of autistic individuals (Case): 31 − Siblings of patients with specific speech articulation disorder (Control): 30	− Case: 7–11 years old − Control: 7–11 years old	− Autoquestionnaire Qualité de Vie Enfant Imagé (AUQEI—Quality of Life of Children aged 4 to 12 years)	Self-reported	− QoL	− The QoL of cases impaired − The scores of the cases were significantly lower than the QoL of controls
Orsmond, G.I. [22]	To investigate sibling relationships and well-being in adolescents and adults with an autistic sibling	− Adolescent siblings of autistic individuals: 56 − Adult siblings of autistic individuals: 142	− Adolescents: 12–18 years old − Adults: 19–55 years old	− Instrumental Involvement—Caregiving − Positive Affect Index (PAI) − Center for Epidemiologic Studies Depression Scale (CES-D)—8 scales from the COPE − Perceived Social Support Scales − Scales of Independent Behavior-Revised (SIB-R)	Self-reported	− Engagement in shared activities − Reported positive affect − Psychological well-being − Coping − Social support	− Stability in the closeness of the sibling relationship when there is one autistic sibling − No group differences in depressive symptoms, in both groups absent − Adolescents used more emotion-focused coping strategies than adults, who used more problem-focused coping strategies − Adolescents reported greater social support than adult siblings − Adult sisters of autistic sisters showed more engagement in shared activities than adult brothers of autistic sisters
Vieira, C.B.M. [31]	To assess the QoL in siblings of autistic children	Siblings of autistic individuals: 21	16–30 years old	− World Health Organization Quality of Life (WHOQoL)-BREF questionnaire	Self-reported	− QoL	− The environment domain had the lowest scores; it was related to the physical and psychological domains − The self-reported QoL for the analyzed sample was not significantly impaired
Hastings, R.P. [32]	To gather sibling self-reports about their behavior problems and to compare these data to a self-report normative sample	− Siblings of autistic individuals (Case): 94 − Children from general population (Control): 4228	− Case: 7–17 years old − Control: 7–17 years old	− Strengths and Difficulties Questionnaire (SDQ) − Sibling Relationship Questionnaire-Revised (SRQ-R)	Self-reported and parent proxy-reported	− Sibling relationship − Sibling adjustment	− No significant elevation in behavioral and emotional problems among siblings of autistic children − Siblings’ relationships may be associated with the behavior problems of the autistic child − Higher behavior problems were associated with lower levels of warmth/closeness and with increased conflict
Chan, J.Y.N. [33]	To explore the psychological adjustment of siblings of autistic children	− Siblings of autistic individuals (Case): 116 − Children from the general population (Control)	− Case: 6–18 years old − Control: 6–18 years old	− Strengths and Difficulties Questionnaire (SDQ) − General Health Questionnaire (GHQ-12) − WHOQoL-BREF − General Functioning Scale (GFS) of the Family Assessment Device (FAD) − Sibling Stress Scale (SSS)	Self-reported and parent proxy-reported	− QoL − Sibling adjustment − Sibling stress − Parental psychological stress − Family functioning	− The total difficulties rates were higher in the reports of parents of autistic children − Siblings’ adjustment was predicted by family stresses − Several endorsed experiences were indicative of a stressful sibling relationship − Parents’ reports ratings were not statistically different between the two groups, except for some concerns about peer relationships and prosocial behaviors among siblings of autistic children
Tomeny, T.S. [34]	To assess adult sibling relationships in families of individuals with a developmental disability (DD)	− Siblings of autistic individuals (Case): 45 − Siblings of patients with developmental disability (ID) (Control): 37	− Case: 18–62 years old − Control: 18–52 years old	− Depression, Anxiety, and Stress Scale (DASS) − Lifespan Sibling Relationship Scale (LSRS) − Satisfaction with Life Scale (SWLS)	Self-reported	− Sibling relationship − Sibling stress − Sibling depressive symptoms − Sibling anxiety − Sibling life satisfaction − Sibling aid	− The siblings of autistic people showed lesser positive attitudes about their relationship with their brother/sister when compared to siblings of people with ID − The siblings of autistic individuals could be at greater risk of developing depression and stress and, consequentially, could provide less support due to fewer positive sibling relationship attitudes
Esfahani, F.N. [35]	To recognize the problems of living with an autistic sibling to improve their QoL	− Siblings of autistic individuals (Case): 30 − Siblings of patients with chronic diseases (Control): 30	− Case: 4–11 years old − Control: 4–11 years old	− Gilliam Autism Rating Scale-Second Edition (GARS-2) − Empathizing-systemizing test − Child Behavior Checklist	Self-reported	− Sibling anxiety − Sibling depression − Sibling empathy − Sibling social problems − Sibling aggressive behavior	− The aggressive behavior of the siblings of autistic individuals was significantly higher than the same item among the siblings of people with chronic disease − The externalizing score was significantly higher in the first group than in the control group − Sisters had higher scores than brothers in terms of anxiety and depression, suggesting different attitudes between females and males
Koukouriki, E. [20]	To investigate for any association between siblings’ health-related QoL (HRQoL) or anxiety and parental psychological health, perceived social support as well as major demographic factors	− Siblings of autistic individuals (Case): 118 − Siblings of non-autistic children (Control): 115	− Case: 8–18 years old − Control: 8–18 years old	− Health-Related QoL (KIDSCREEN-27) − State-Trait Anxiety Inventory for Children (STAIC, A-Trait) − General Health Questionnaire (GHQ-28) − Multidimensional Scale of Perceived Social Support (MSPSS)	Self-reported and parent proxy-reported	− HRQoL − Sibling anxiety − Family perceived social support − Parental psychological health	− After controlling for sex and age of the children, the HRQoL of the siblings of autistic individuals was lower than in the control group − Anxiety levels of the case group were significantly higher than in the control group − Perceived social support from the family was a predictor of HRQoL of the siblings of autistic children − The case group had lower scores than the control group in each subscale of KIDSCREEN-27, with the highest impairment in the subscale of psychological well-being − The parents of the cases group showed higher levels of psychological distress than the control group − The hierarchical regression models showed that social support from the family was associated with siblings’ HRQoL, and that the anxiety levels of siblings of autistic children were associated with parental anxiety
Garrido, D. [9]	To explore potential factors that help explain the impact of having an older autistic sibling on several developmental domains and to test whether these factors could explain their satisfaction on family QoL (FQoL)	− Siblings of autistic individuals (Case): 41 − Siblings of non-autistic children (Control): 37	− Case: 6–12 years old − Control: 6–12 years old	− Comprehension test of grammatical structures (CEG) − Peabody Picture Vocabulary Test (PPVT-III) − Clinical Evaluation of Language Fundamentals-Fourth Edition (CELF-4) − Movement Assessment Battery for Children-Second Edition (MABC-2) − Wechsler Intelligence Scale for Children (WISC-IV) − Social Communication Questionnaire (SCQ) − Structural Social Support − Family QoL of People Survey (FQoLS)	Self-reported and parent proxy-reported	− FQoL − Sibling perceived social support − Sibling language − Sibling motor skills − Sibling intelligence − Sibling social communication	− There were statistically significant differences in the severity of the autism spectrum, FQoL scores, and perceived social support between the group of siblings of autistic people and the siblings of typically developing children − Perceived social support might be a predictor of FQoL: the higher the social support is perceived, the higher the quality of life

## Data Availability

Not applicable.
